# Hidden Sylvatic Foci of the Main Vector of Chagas Disease *Triatoma infestans*: Threats to the Vector Elimination Campaign?

**DOI:** 10.1371/journal.pntd.0001365

**Published:** 2011-10-25

**Authors:** Leonardo A. Ceballos, Romina V. Piccinali, Paula L. Marcet, Gonzalo M. Vazquez-Prokopec, M. Victoria Cardinal, Judith Schachter-Broide, Jean-Pierre Dujardin, Ellen M. Dotson, Uriel Kitron, Ricardo E. Gürtler

**Affiliations:** 1 Laboratory of Eco-Epidemiology, Department of Ecology, Genetics and Evolution, Universidad de Buenos Aires, Buenos Aires, Argentina; 2 Centers for Disease Control and Prevention, Division of Parasitic Diseases and Malaria, Atlanta, Georgia, United States of America; 3 Department of Environmental Studies, Emory University, Atlanta, Georgia, United States of America; 4 Fogarty International Center, National Institutes of Health, Bethesda, Maryland, United States of America; 5 Unité Mixte de Recherche, Institut de Recherches pour le Développment-Centre National de Recherche Scientifique, Montpellier, France; Universidad Centroamericana, Nicaragua

## Abstract

**Background:**

Establishing the sources of reinfestation after residual insecticide spraying is crucial for vector elimination programs. *Triatoma infestans*, traditionally considered to be limited to domestic or peridomestic (abbreviated as D/PD) habitats throughout most of its range, is the target of an elimination program that has achieved limited success in the Gran Chaco region in South America.

**Methodology/Principal Findings:**

During a two-year period we conducted semi-annual searches for triatomine bugs in every D/PD site and surrounding sylvatic habitats after full-coverage spraying of pyrethroid insecticides of all houses in a well-defined rural area in northwestern Argentina. We found six low-density sylvatic foci with 24 *T. infestans* in fallen or standing trees located 110–2,300 m from the nearest house or infested D/PD site detected after insecticide spraying, when house infestations were rare. Analysis of two mitochondrial gene fragments of 20 sylvatic specimens confirmed their species identity as *T. infestans* and showed that their composite haplotypes were the same as or closely related to D/PD haplotypes. Population studies with 10 polymorphic microsatellite loci and wing geometric morphometry consistently indicated the occurrence of unrestricted gene flow between local D/PD and sylvatic populations. Mitochondrial DNA and microsatellite sibship analyses in the most abundant sylvatic colony revealed descendents from five different females. Spatial analysis showed a significant association between two sylvatic foci and the nearest D/PD bug population found before insecticide spraying.

**Conclusions:**

Our study shows that, despite of its high degree of domesticity, *T. infestans* has sylvatic colonies with normal chromatic characters (not melanic morphs) highly connected to D/PD conspecifics in the Argentinean Chaco. Sylvatic habitats may provide a transient or permanent refuge after control interventions, and function as sources for D/PD reinfestation. The occurrence of sylvatic foci of *T. infestans* in the Gran Chaco may pose additional threats to ongoing vector elimination efforts.

## Author Summary

*Triatoma infestans*, a highly domesticated species and historically the main vector of *Trypanosoma cruzi*, is the target of an insecticide-based elimination program in the southern cone countries of South America since 1991. Only limited success has been achieved in the Gran Chaco region due to repeated reinfestations. We conducted full-coverage spraying of pyrethroid insecticides of all houses in a well-defined rural area in northwestern Argentina, followed by intense monitoring of house reinfestation and searches for triatomine bugs in sylvatic habitats during the next two years, to establish the putative sources of new bug colonies. We found low-density sylvatic foci of *T. infestans* in trees located within the species' flight range from the nearest infested house detected before control interventions. Using multiple methods (fine-resolution satellite imagery, geographic information systems, spatial statistics, genetic markers and wing geometric morphometry), we corroborated the species identity of the sylvatic bugs as *T. infestans* and found they were indistinguishable from or closely related to local domestic or peridomestic bug populations. Two sylvatic foci were spatially associated to the nearest peridomestic bug populations found before interventions. Sylvatic habitats harbor hidden foci of *T. infestans* that may represent a threat to vector suppression attempts.

## Introduction

Disease eradication or elimination programs depend on time-limited intensive campaigns and are likely to fail if resistance to insecticides or drugs (i.e., malaria) or sylvatic transmission cycles (i.e., yellow fever) occur. Chagas disease is the most important vector-borne disease in Latin America in terms of disability-adjusted lost years, with an estimated 10–18 million people infected with *Trypanosoma cruzi* [1]. Elimination of domestic or peridomestic (hereafter abbreviated D/PD) populations of the insect vectors of *T. cruzi* through residual spraying with insecticides has shown varying degrees of success depending on the species and the occurrence of sylvatic foci. Several vector species occupy sylvatic habitats and show different degrees of domestication, such as *T. dimidiata* in Central America, *Panstrongylus megistus*, *T. brasiliensis* and *T. pseudomaculata* in Brazil, *Rhodnius ecuadoriensis* in northern Peru and Ecuador, and *T. pallidipennis* and related species in Mexico [2]–[4]. Species of sylvatic or peridomestic triatomines that were not recognized as control targets have emerged as primary vectors of *T. cruzi* in geographically defined areas over the last two decades [e.g., 5]. For species such as *R. prolixus*, house reinfestations may also be driven by invasion from peridomestic or sylvatic foci [6].

*Triatoma infestans* historically is the main vector of human *T. cruzi* infection. In 1991, this species was the target of a regional elimination program (the Southern Cone Initiative) that interrupted vector- and blood-borne transmission to humans in Chile, Uruguay, Brazil, eastern Paraguay and parts of Argentina [7]. However, only limited success in the elimination of *T. infestans* and interruption of vector-borne transmission has been achieved in the Gran Chaco region due to repeated reinfestations even in areas under intensive professional vector control [8]. The Gran Chaco, an ecoregion of 1.3 million km^2^ mainly spanning northern Argentina, Bolivia and Paraguay, has high levels of poverty and is hyperendemic for Chagas disease [9]. Recurrent reinfestation after residual spraying with insecticides and lack of a sustainable vector surveillance program result in renewed parasite transmission 3–5 years after community-wide vector control campaigns [10]–[13]. The obstacles to the elimination of *T. infestans* in the Gran Chaco may stem from different processes yet to be identified conclusively.

The Southern Cone Initiative for the elimination of *T. infestans* was based on two major assumptions with wide consensus and limited supporting evidence [14], [15]: (i) the species was restricted to D/PD habitats [16]–[19], with true sylvatic foci only occurring in rock piles associated with wild guinea pigs in the Cochabamba and Sucre Andean valleys in Bolivia [20]–[22], and (ii) *T. infestans* had low genetic variability and therefore was very unlikely to develop resistance to modern pyrethroid insecticides. Rare findings of *T. infestans* in sylvatic habitats up to the early 1980 s were judged to be of little relevance by several investigators (reviewed in [23], [24]). The surprising finding of melanic forms (“dark morphs”) in isolated dry forests in the Bolivian [23], [25] and Argentine Chaco [24], and more recently in the Paraguayan Chaco [26], combined with the discovery of sylvatic foci with normal phenotypes in Chile and Bolivia [27]–[29] challenged the highly domesticated status of *T. infestans*. In addition, recent evidence showed *T. infestans* had richer genetic variability than previously assumed [30]–[33], with strong chromosomal and DNA content differences between *T. infestans* from different sources [34], whereas pyrethroid resistance emerged in northwestern Argentina and throughout Bolivia since the late 1990 s [35], [36]. Understanding the ecological dynamics of reinfestation in insecticide-treated villages and untangling the mechanisms underlying the observed patterns is crucial for devising improved vector control tactics and the eventual elimination of *T. infestans* and other major triatomine vectors [18], [37]. Genetic [38] and phenetic [39], markers combined with carefully georeferenced bug samples collected before and after control interventions, a geographic information system (GIS) and spatial statistics [41] provide the means to better understand reinfestation dynamics. Here we first integrate the use of all these tools to investigate house reinfestation dynamics in the context of control interventions.

As part of a longitudinal project on the eco-epidemiology and control of Chagas disease in a well-defined rural area in the dry Argentine Chaco [8], we detected isolated findings of adult *T. infestans* and recently established, very low-density D/PD colonies during two years after a community-wide residual spraying of pyrethroid insecticides of all houses. To identify the putative sources for such occurrences and the sylvatic vectors of *T. cruzi* [42], we conducted intensive surveys for triatomine bugs in diverse sylvatic habitats after interventions and surprisingly found various sylvatic foci of *T. infestans*. Using fine-resolution satellite imagery, GIS, spatial statistics, genetic markers and wing geometric morphometry, we investigated the relatedness between sylvatic and D/PD populations of *T. infestans* and the threat that they may represent to vector control and elimination attempts in the Argentinean Chaco. Based on previous findings of sylvatic *T. infestans* in the Bolivian Chaco [43] and of an isolated adult specimen of *T. infestans* infected with *T. cruzi* in semi-sylvatic habitats of our study area in the mid-1980 s [44], we speculated that similar foci might exist in the Argentinean Chaco and that *Triatoma guasayana* was a likely candidate sylvatic vector of *T. cruzi* given its high abundance, widespread occurrence and occasional infection with the parasite [43], [45], [46].

## Materials and Methods

### Study area

**Figure 1:**
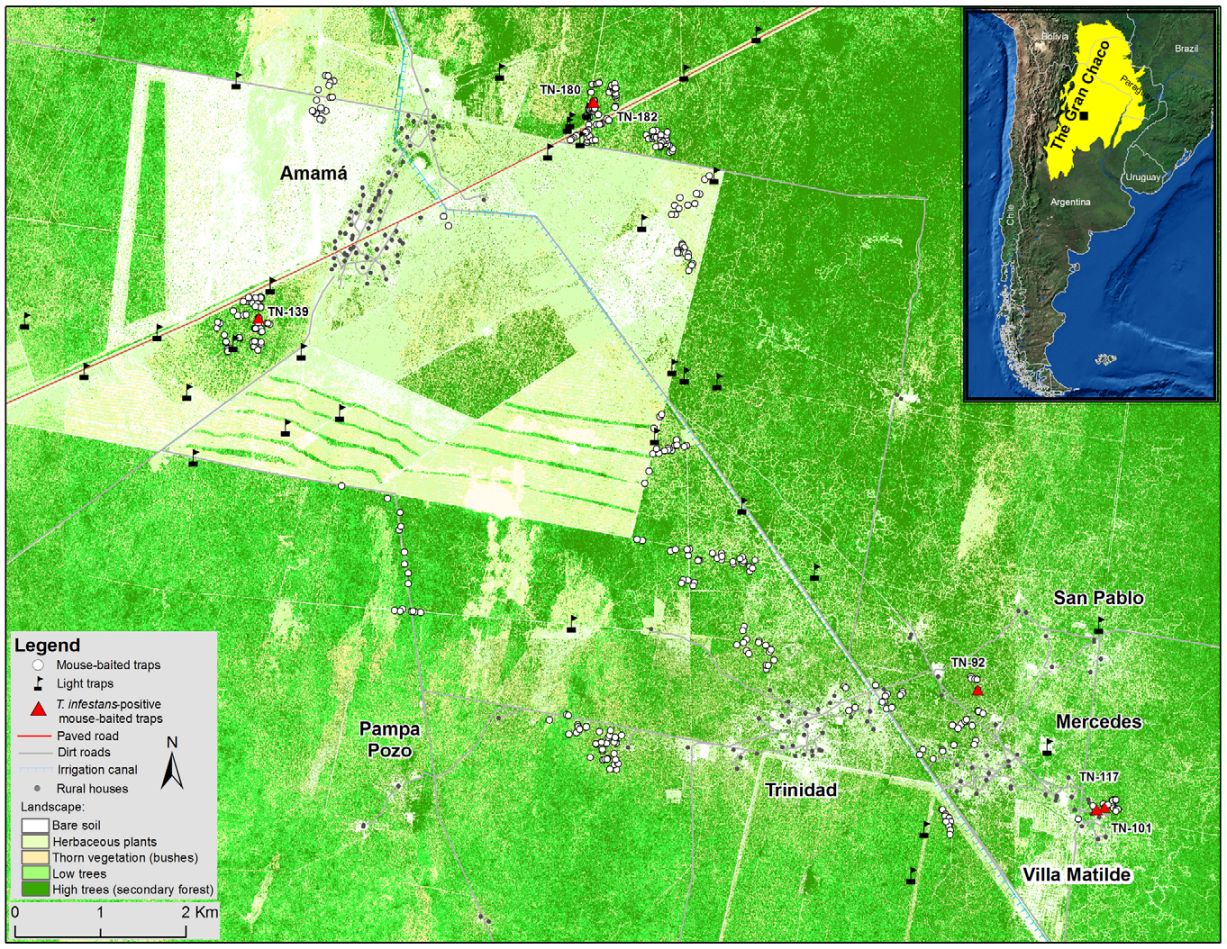
Map of the study area indicating the position of mouse-baited and light traps. Red triangles indicate the position of *T. infestans*-positive mouse-baited traps. Inset shows the location of the study area (black square) within the Gran Chaco region.

Field studies were carried out in Amamá (27° 12′ 30″S, 63° 02′ 30″W) and neighboring rural villages in a 650 km^2^ area situated in the Moreno Department, Province of Santiago del Estero, Argentina (Figure 1). This area is located in the dry Chaco ecoregion [42] and its history of infestation since the mid-1980 s has been described elsewhere [8]. Based on the history of control interventions, the study area was subdivided into core (5 villages, 143 domiciles and 790 peridomestic sites) and peripheral (7 villages, 132 houses and 709 peridomestic sites) areas with all sites georeferenced. In April 2004, community-wide residual spraying with 2.5% deltamethrin (K-Othrin, Bayer) of nearly all houses was conducted by professional vector-control personnel using a standard insecticide dose in domiciles (25 mg/m^2^) and standard or double dose in peridomestic sites for enhanced impact. Here we only report results from the core area (villages of Amamá, Trinidad, Mercedes, Villa Matilde and Pampa Pozo; Figure 1) because no systematic searches for bugs were performed in sylvatic habitats around the peripheral communities.

### Vector collection

Timed manual searches for triatomine bugs with a dislodging spray (0.2% tetramethrin, Espacial) were conducted in all domestic (0.5 person-hour) and peridomestic sites (one person-hour per house compound) from all study villages in October 2004, April and December 2005, and November 2006 as described before [10]. In the core area, 143 domiciles and 764 peridomestic sites were inspected for triatomine bugs at least once between 2004 and 2006. All detected foci were immediately sprayed with deltamethrin using the same procedures. As part of an ongoing monitoring program, discriminant dose assays demonstrated that no pyrethroid resistance occurred in local populations of *T. infestans* (María Inés Picollo, unpublished results).

We conducted four intensive surveys of triatomine bugs in sylvatic habitats using mouse-baited (Noireau) traps fitted with adhesive tape (Plasto®, Brazil) [47] in October and November 2005, April and November-December 2006 as described before [24]. Mean temperatures varied between 23°C and 26°C in October-December (spring) surveys, and were below 20°C in April (fall). Searches for sylvatic triatomine foci were conducted in 15 sampling areas that included representative forest sections with different degrees of disturbance (i.e., degraded forest under logging operations, cleared sections, ecotones, and implanted grasslands preceded by selective deforestation) and in all sorts of refuges potentially suitable for triatomine bugs. The total capture effort was 598 trap-nights (range per survey, 129 to 169). Traps were usually placed far from houses in holes of fallen or standing trees (live or dead), trunks or tree stumps and in between terrestrial bromeliads (*Bromelia serra* and *Bromelia hieronymi*), cacti (*Opuntia quimilo* and *Opuntia ficus-indica*) or piles of shrubs (Figure S1). Traps were deployed when the weather was warm and not rainy approximately between 17.00–18.00 hs and retrieved before 10.00 hs to protect mice from exposure to extreme temperatures. All trap locations were georeferenced using a GPS (Garmin, Etrex Legend C). All sylvatic sites surveyed in October and November 2005 were different except one, and 98% of them were re-inspected with mouse-baited traps on April 2006 to assess bug occurrence, persistence and invasion. The survey conducted in November-December 2006 only included sites that had not been surveyed previously.

Flight-dispersing triatomine bugs were collected using black-light traps [48] placed in 36 georeferenced sylvatic sites where concurrent searches with mouse-baited traps were made (i.e., in the same areas). Light traps were deployed away from houses in habitats where there was a wide opening in the forest that allowed at least a 100 m visibility. Light traps were operated from approximately 19:45 (i.e., 15 min before sunset) to 22:00–23:00 hs because the flight activity of *T. infestans* peaks during the first hour after sunset, and is more likely to occur when air temperature exceeds 20°C and wind speed is <5 km/h [48]–[50]. Suitable conditions for flight initiation of *T. infestans* occurred during the surveys conducted in October-November 2005 but not in April 2006.

All collected bugs were kept alive in plastic vials with folded filter paper, identified to species following Lent and Wygodzinsky [19] and counted. Species identification of very small first- or second-instar nymphs sometimes was considered tentative depending on the integrity of the material; no such doubts remained for third-instars or later stages. Fourth- or fifth-instar nymphs and adult bugs collected in 2005 were individually weighed on an electronic balance (OHAUS, precision, 0.1 mg) and total body length (L) measured from the end of the clipeus to the end of the abdomen with a vernier caliber (precision, 0.02 mm) to estimate a weight-to-length ratio (W∶L) –a quantitative index of nutritional status. The qualitative nutritional status of nymphs was determined by a cross-sectional view of the abdomen and cuticle distension and classified into four categories that ranged from unfed to large blood contents [51]. Feces from live third-instars and larger stages were examined microscopically for *T. cruzi* infection at 400× magnification.

### Genetic characterization

DNA from bugs assigned to *T. infestans* (based on morphological evidence) was obtained, PCR-amplified, and sequenced for a 661 bp fragment of the mitochondrial genes cytochrome oxidase I (mtCOI) [32] and a 572 bp fragment of the cytochrome B (mtcytB) gene [52]. Sequences from sylvatic *T. infestans* were compared with *Triatoma* spp sequences available at Genbank and from previous surveys on the instraspecific variability of *T. infestans* [32], [53]–[56].

Sylvatic *T. infestans* mtCOI plus mtcytB composite haplotypes were compared with previously recorded haplotypes of D/PD *T. infestans* from the study villages (collected in 2001–2002), from other more distant (40 km) localities within Santiago del Estero Province (Quilumpa, Km 40, La Loma and Invernada Norte, collected in 2003–2004), and from other Argentinean Provinces more than 300 km apart (Salta, La Rioja, Tucumán and Formosa, collected in 2000–2005). A detailed description of the source localities was published elsewhere [32]. Genetic variability was estimated as the mean number of pairwise differences per site (π), Watterson's estimator (θ_W_) and the haplotype diversity (Hd) with DnaSP 5.0 [57] and a statistical parsimony haplotype network was built with TCS 1.21 [58].

For higher resolution of the relationships between sylvatic and D/PD populations of *T. infestans*, the multilocus (ML) genotype for 10 microsatellite loci was obtained for sylvatic *T. infestans* using primers and PCR conditions previously described [59]. ML genotypes were compared with those from *T. infestans* captured in D/PD sites from Amamá and neighboring villages in October 2002 and April 2004 before full-coverage insecticide spraying [60]. Inter-individual genetic distance based on the complement of the proportion of shared alleles [61] was estimated with MICROSAT 1.5d (http://hpgl.stanford.edu/projects/microsat/), and a neighbor-joining (NJ) tree was built with MEGA 3.0 [62].

Using the genotypes of local D/PD *T. infestans* as reference populations, we applied the Bayesian based assignment-exclusion test implemented in GENECLASS 2 [63] to individually assign sylvatic individuals to the local pre-spraying D/PD populations (defined as the total gene pool at a given community in each capture date). No post-spraying reference groups could be formed because after community-wide insecticide spraying (2004–2006) most bug collections contained one or a few insects per site that were sparsely distributed throughout the communities (i.e., no established populations of *T. infestans* were detected). Reference populations were not excluded as the putative origin of the sylvatic insects when the marginal probability exceeded 0.05. We used 100,000 replications and a simulation algorithm [64].

Sibship of *T. infestans* bugs collected in traps with more than one individual (TN-92 and TN-139) was inferred with the maximum likelihood approach implemented in COLONY 2.0 [65] performing two independent runs and assuming a probability of null alleles of 0.05 in loci ms42, ms64 and ms65 due to departures from Hardy-Weinberg expectations.

### Geometric morphometry

The wing geometric morphometry of the only sylvatic *T. infestans* male collected was compared with *T. infestans* males captured in D/PD sites from Amamá and neighboring study villages in October 2002 (n = 87) and April 2004 (n = 74) as described elsewhere [66]. The geometric coordinates of 11 type-I landmarks (venation intersections) from all right wings were digitized by the same user (JSB). After performing the generalized Procrustes superposition (GPA, [67]), the residual coordinates of the total sample (including the sylvatic specimen) were transformed into partial warps (PW). These shape variables allow standard statistical analyses such as principal component (PCA) or discriminant analyses (DA). To cope with small sample sizes in some villages, the first nine principal components of the PW were used as input for a DA performed on the village samples (excluding the sylvatic specimen). These principal components are also called relative warps (RW). The sylvatic specimen was then used as supplementary data and its position in the morphospace examined in terms of Mahalanobis distances. Digitization, GPA, PCA and DA were performed using the corresponding modules of the CLIC package [68].

### Spatial analysis

Global positioning system readings from all sampling sites (with mouse-baited and light traps) were integrated into a Geographic Information System (ArcGIS 9.1, ESRI, Redlands, CA, U.S.A.) of the study communities containing a georeferenced satellite image (Ikonos2, Space Imaging Inc., Atlanta, GA, U.S.A.) and the position of all houses and peridomestic sites sprayed with insecticides in 2004. Cartesian coordinates (Universal Transverse Mercator, UTM, Zone 20S) were calculated for each D/PD site and trapping location in order to perform spatial analysis. A focal spatial statistic (G_i_(d)) [69] was used to determine the presence and extent of spatial clustering of *T. infestans* D/PD abundance (average of timed manual catches of bugs per site in 2002 and 2004] around each *T. infestans*-positive sylvatic focus (point *i*). This local statistic is additive in the sense that it focuses on the sum of the *j* values in the vicinity of point *i*. Hence, we took each *T. infestans*-positive sylvatic focus, one at a time, and searched the nearby area for occurrences of more or fewer D/PD *T. infestans* bugs collected before full-coverage insecticide spraying than expected by random. This procedure identified specific trap locations as members or non-members of infestation clusters. We used a binary weight *w_ij_* based on a distance threshold (*d*) scheme. Clustering of D/PD *T. infestans* abundance around a positive sylvatic site occurred when the observed *G_i_* was higher than 2.32 (the expected value at *P*<0.01). We evaluated the value of *G_i_* up to 3 km from each sylvatic site with *T. infestans* –a tentative upper bound of the flight range of *T. infestans*. Analyses were performed using the software Point Pattern Analysis [70].

### Ethics Statement

Humane care and use of laboratory animals were performed according to Institutional Animal Care and Use Committee (IACUC, CICUAL in Spanish) guidelines at UBA's Faculty of Exact and Natural Sciences. Animal care and use is guided by the International Guiding Principles for Biomedical Research Involving Animals developed by the Council for International Organizations of Medical Sciences.

## Results

### Collection and nutritional state of triatomine bugs

A total of 13 (9.1% of 143 domiciles) domestic foci of *T. infestans* with 23 bugs and 38 (5.0% of 764 sites) peridomestic foci with 223 bugs were detected between 2004 and 2006 after full-coverage spraying with deltamethrin. Nearly 25% of all collected *T. infestans* were adult bugs.

Only 30 (5%) of 598 mouse-baited traps deployed overnight in sylvatic habitats were positive for triatomine bugs (Table 1). Six sylvatic foci of *T. infestans* with normal chromatic characters (totaling 23 nymphs and 1 male; range per site, 1–17) were found in tree holes or trunks (Figures S1 and S2). Another probable sylvatic foci of this species with two first- or second-instar nymphs was conservatively excluded because the morphological identification of these stages was uncertain and mtDNA markers did not amplify; this probable focus occurred in the vicinity of the largest sylvatic colony of *T. infestans* (trap TN-139, Figure S2). The apparent density of sylvatic *T. infestans* was 4 per 100 trap-nights (24 bugs in 598 trap-nights; mean ± SD, 3.8±6.4 bugs per site). One sylvatic focus located west of Amamá (trap TN-139) was infested both in October (1 male) and November 2005 (14 first- or second-instar nymphs and 2 fourth-instars) and was taken as one colony. No *T. infestans* bugs were collected with mouse-baited traps in April or November 2006.

**Table 1:** Occurrence and relative abundance of *T. infestans*, *T. guasayana* and other triatomine in sylvatic habitats.

Capture method	Survey	No. trap-nights	% positive traps (No. bugs collected)			
			*T. infestans*	*T. guasayana*	Other *Triatoma sp.*	Total
Mouse-baited traps	October 2005	145	2.8 (6)	1.4 (2)	0 (0)	4.1 (8)
	November 2005	129	2.3 (18)	2.3 (6)	2.3 (7)^a^	7.0 (31)
	April 2006	155	0 (0)	8.4 (20)	0 (0)	8.4 (20)
	Nov–Dec 2006	169	0 (0)	0 (0)	1.2 (3)^a^	1.2 (3)
	Total	598	1.2 (24)	3.0 (28)	0.8 (10)	5.0 (62)
Light traps	October 2005	18	0 (0)	72.2 (70)	7.7 (1)#	72.2 (71)
	November 2005	19	0 (0)	68.4 (35)	7.7 (1)&	68.4 (36)
	Nov–Dec 2006	4	0 (0)	50.0 (5)	0 (0)	50.0 (5)
	Total	41	0 (0)	68.3 (110)	4.9 (2)	68.3 (112)

a First- or second-instar nymphs, probably *T. guasayana*.

# *T. garciabesi*.

& *T. platensis*.

Amamá and neighboring villages, 2005–2006.

*T. guasayana* occurred more frequently (3.0% of mouse-baited traps in all surveyed habitats) than *T. infestans* (1.2%, Table 1). Feces and hairs of *Didelphis* opossums were found in one *T. guasayana* focus. All first- or second-instars of *Triatoma sp*. not identified to species level most likely were *T. guasayana* based on morphology, size and type of habitat. Light-trap collections yielded 110 adult *T. guasayana*, one specimen of *T. garciabesi* (female) and one of *T. platensis* (male), and no *T. infestans* in 41 light-trap-nights (Table 1). Of the 41 light-trap nights, 28 (68.3%) were positive for triatomine bugs. The adult sex ratio in *T. guasayana* was 1∶2.2 (male to female).

**Figure 2:**
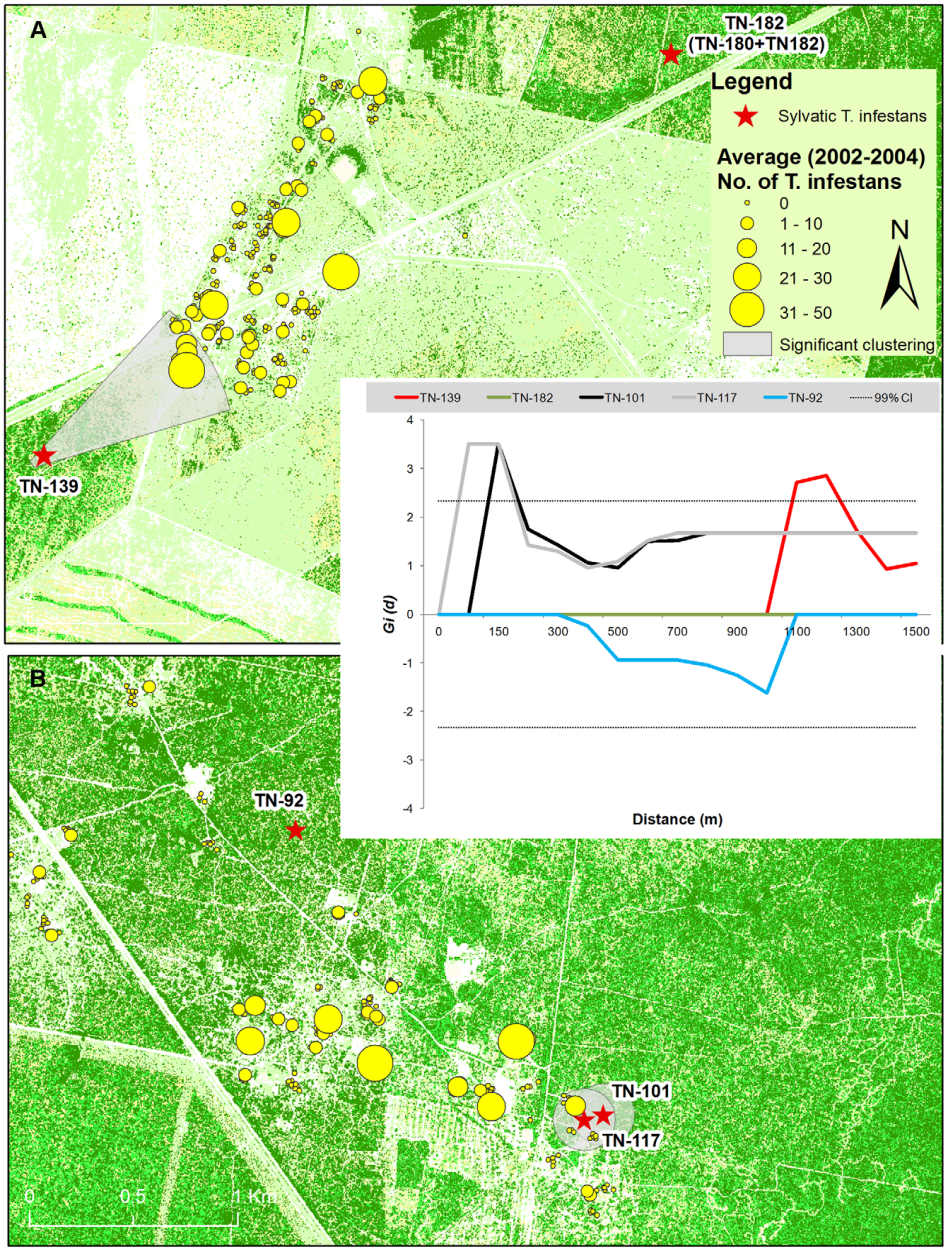
Spatial association between sylvatic and peridomestic *T. infestans* colonies. Plot of (*G_i_(d)*) values estimated for the peridomestic abundance of *T. infestans* (2002–2004) as a function of distance to each sylvatic focus within 3 km of Amamá (A) and Mercedes (B). Dotted lines represent 99% confidence intervals.

Sylvatic foci of *T. infestans* occurred at 5 sampling areas located 2.0–11.5 km apart (Figure 2). Most triatomines (17 or 70.8% of 24 *T. infestans* and 18 or 64.3% of 28 *T. guasayana*) caught with mouse-baited traps occurred in areas that had been deforested selectively (totalling 40 bugs at 11 sites); the other seven *T. infestans* were caught in secondary forest with medium-sized or a few large-sized trees. The only three *T. garciabesi* found were caught in mature forest under active deforestation. The remaining triatomine bugs were caught in secondary forest with medium- or large-sized trees. The main identified micro-habitats of *T. infestans* were in holes of fallen trees and decaying tree trunks lying on the ground (21 or 87% of 24 bugs collected), a tree stump and a live standing tree. These ecotopes included 4 ‘quebracho colorado’ (*Schinopsis lorentzii*) and 2 ‘mistol’ (*Zizyphus mistol*) trees (Figure S1).

Nearly all triatomine bugs caught with mouse-baited traps and examined for qualitative nutritional status (n = 36) were unfed (61.1%) or had very little remnants of a blood meal (33.3%) and very low W/L ratios (Table S1). Of 140 sylvatic triatomine bugs examined microscopically (10 *T. infestans*, 21 *T. guasayana* and 3 *T. garciabesi* caught with mouse-baited traps and 106 *T. guasayana* collected with light traps) none was found microscope-positive for *T. cruzi*.

### mtDNA analyses of *T. infestans*

The morphological identification of 20 sylvatic bugs as *T. infestans* was confirmed by DNA sequencing of mtCOI and/or mtcytB fragments; DNA from six other bugs (all first- to third-instars identified as *T. infestans* based on morphological characters) could not be amplified. The two third-instar nymphs not amplified were taken as *T. infestans* because a morphological misidentification (relative to the locally known species) was considered very unlikely. None of the sylvatic *T. infestans* bugs carried the T_C change at position 556, which is characteristic of *T. platensis* and is absent in a large sample of *T. infestans* from Argentina, Bolivia, Peru, and Uruguay [32].

**Figure 3:**
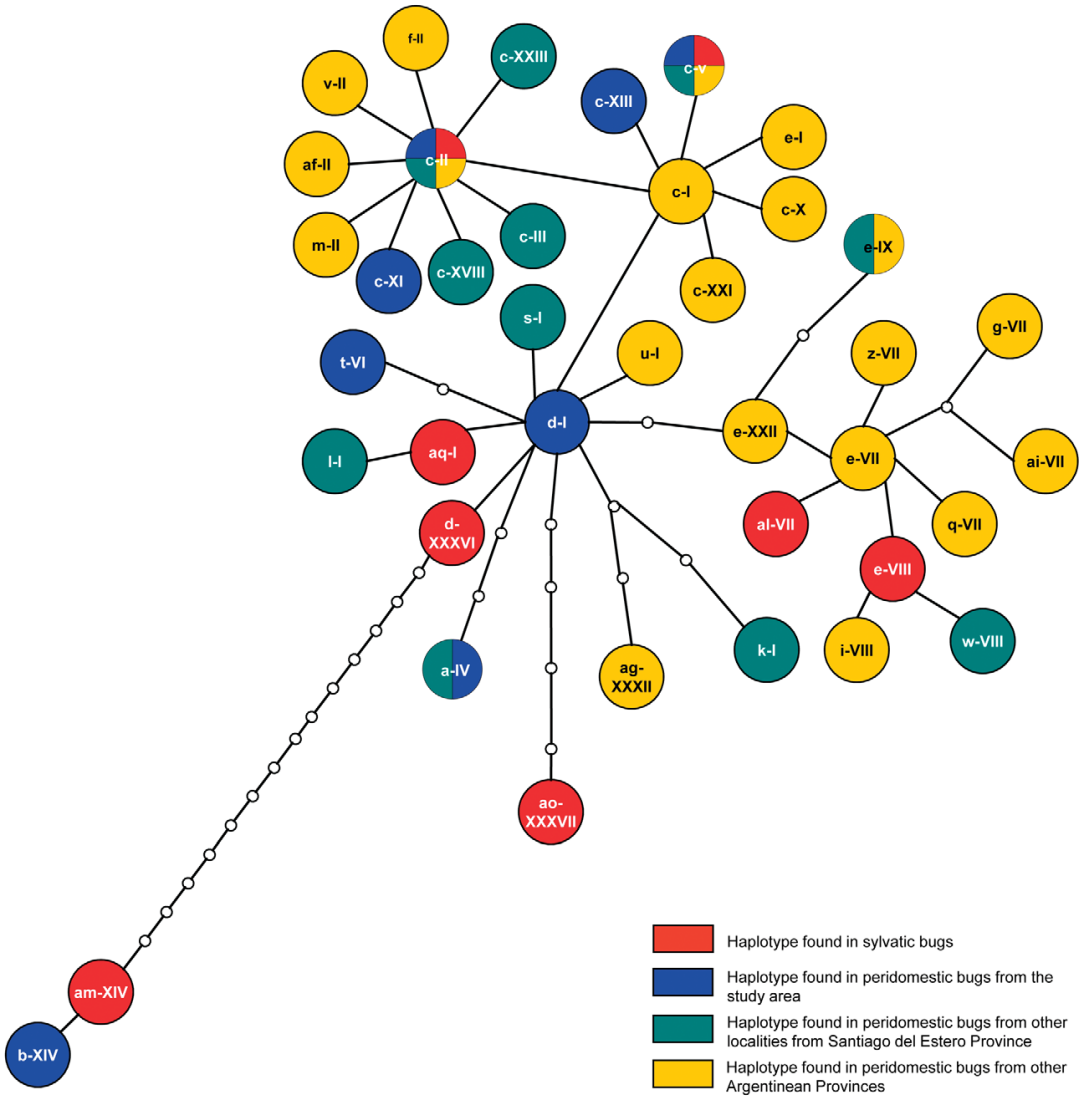
Statistical parsimony network of the composite mitochondrial haplotypes (mtCOI – mtcytB). Each line represents a mutational step and the small empty circles are unobserved haplotypes.

Sylvatic *T. infestans* with mtCOI and mtcytB composite haplotypes (n = 16, Table S2) exhibited high nucleotide variability (θ_W_ = 0.006, π = 0.007) and haplotype diversity (Hd = 0.901). No shared haplotypes were found among bugs from different traps, whereas traps with more than one bug had one (TN-92, n = 3) and five (TN-139, n = 11) different haplotypes (Table S2). Of eight sylvatic haplotypes identified, six were exclusive of sylvatic bugs whereas two haplotypes were recorded in local peridomestic populations of *T. infestans* and elsewhere in Argentina (Figure 3). Sylvatic haplotypes were spread along the entire statistical parsimony network; they did not form a unique cluster separated from the rest and were more closely related to D/PD than to other sylvatic haplotypes (Figure 3). One sylvatic haplotype was highly divergent (am-XIV) but also was closely connected to an Amamá peridomestic haplotype (haplotype b-XIV).

### Microsatellite and wing morphometry analyses

**Figure 4:**
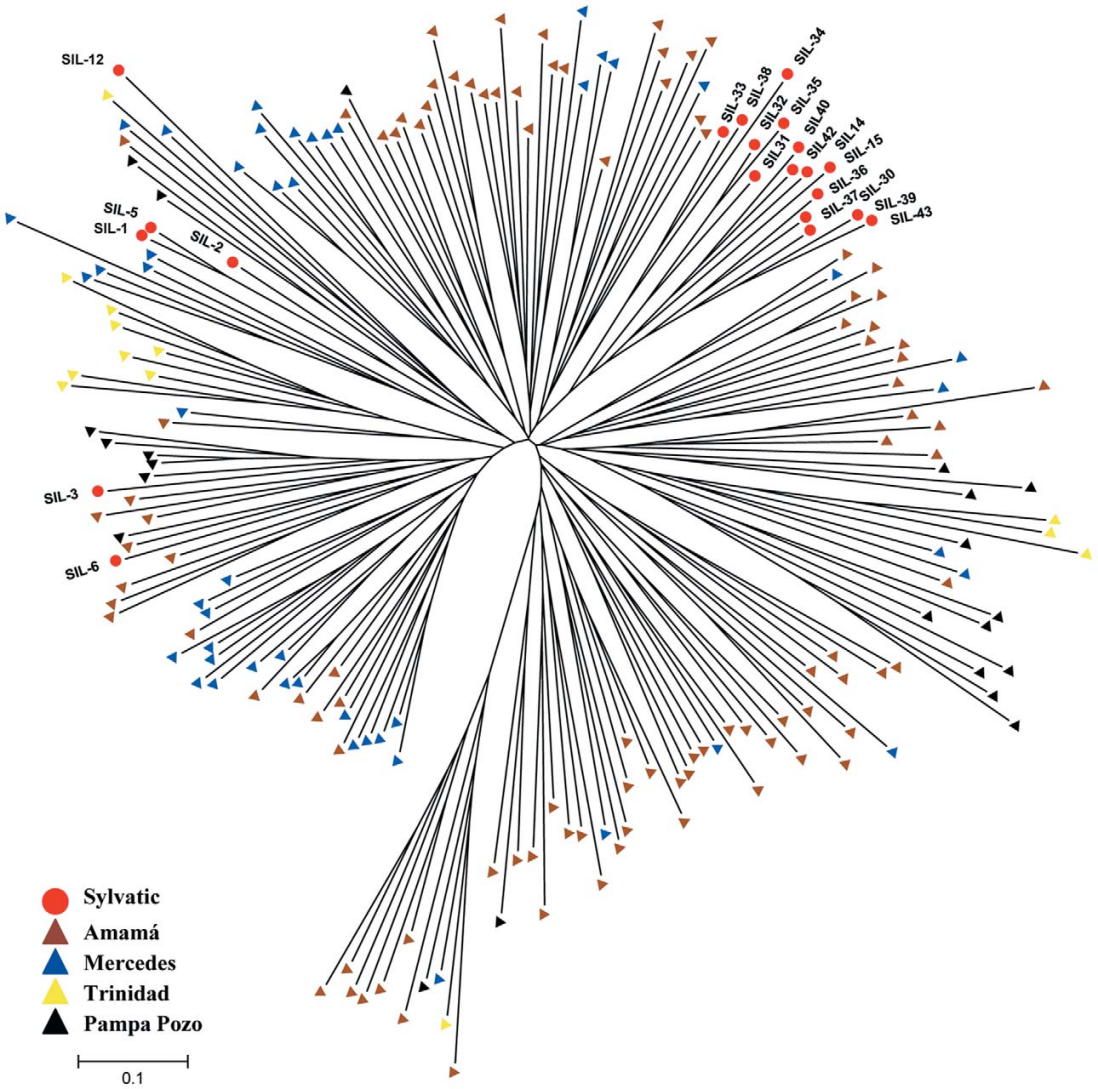
NJ unrooted tree among individuals based on the proportion of shared alleles. Comparison of sylvatic with domestic or peridomestic *T. infestans* populations captured before full-coverage insecticide spraying in 2002.

The multilocus (ML) genotype for 10 microsatellite loci was obtained for 21 sylvatic *T. infestans*. We identified a total of 86 different alleles for the 10 loci, of which only 15 (17.5%) and 17 (19.8%) were private alleles not detected in the local D/PD populations in 2002 and 2004, respectively. Sylvatic *T. infestans* clustered among D/PD bugs with no sharp discontinuity (Figure 4). *T. infestans* bugs captured concurrently at trap TN-139 clustered together whereas bugs collected there at different times were more closely related to different clusters of Amamá peridomestic bugs (i.e., the closest village). In addition, insects from trap TN-139 had five different mtCOI-mtcytB haplotypes (Table S2). Sibship microsatellite analyses showed that bugs that shared a mitochondrial haplotype (or that had consistent haplotypes because of missing data for mtCOI or mtcytB) were most likely full- or half-sibs whereas bugs with different haplotypes were not (Tables S3 and S4). Bugs from trap TN-92 clustered together and closely to bugs from Mercedes village (where the trap was located) and from another village at ∼5 km (Pampa Pozo). These three bugs were full- or half-sibs and shared the same mitochondrial haplotype (Tables S3 and S4). The bug from site trap TN-182 was grouped with bugs from the nearest village (Mercedes) located at ∼8 km. The bug collected at trap TN-101 (close to Villa Matilde, Fig. 1) clustered with bugs from Amamá and Pampa Pozo.

The Bayesian-based assignment-exclusion test indicated that 18 of 21 sylvatic ML genotypes were not excluded from one or more of the D/PD reference populations (Table 2). D/PD populations were excluded as putative sources for three sylvatic insects captured in two different sites (traps TN-182 and -139). The mtCOI-mtcytB haplotype from the bug in trap TN-182 (al VII, Figure 4) was also genetically distant from the local D/PD populations and was closely related to D/PD populations from La Rioja, more than 400 km far from the study area (Figure 4).

**Table 2:** Individual assignment/exclusion results based on Bayesian algorithms tests.

Reference populations: gene pool at communities in a given capture date									
Insect ID	Capture site	A2002	A2004	M2002	M2004	PP2002	PP2004	T2002	T2004
SIL-1	TN-92 A	0.433	0.159	**0.669**	**0.595**	0.074			0.070
SIL-2		0.886	0.821	**0.902**	**0.906**	**0.982**	0.109		0.863
SIL-5		0.260		**0.336**	0.189	0.203			0.063
SIL-3	TN-101	0.094		0.073		0.316			
SIL-12	TN-182	not assigned							
SIL-6	TN-139	**0.123**							
SIL-14		**0.114**	0.100		0.079				
SIL-15		not assigned							
SIL-30		**0.178**				0.108			
SIL-31		0.187		0.154	**0.261**	0.178			
SIL-32		0.154				**0.494**			
SIL-33		0.136		**0.184**	0.116	**0.487**		0.102	0.089
SIL-34					0.060				
SIL-35		0.121				**0.465**			
SIL-36					0.101				
SIL-37		**0.269**	0.126	0.079	0.068				
SIL-38		**0.226**	0.167		**0.244**				0.131
SIL-39		0.118				**0.371**			
SIL-40		0.139							
SIL-42		0.106			0.050				
SIL-43		not assigned							

The numbers in the table are the probabilities of assigning each ML genotype to the reference populations of *T. infestans*. Only inclusion values with P>0.05 are reported. A2002, Amamá in 2002: M2002, Mercedes in 2002; PP2002, Pampa Pozo in 2002; T2002, Trinidad in 2002, and similar symbols for populations in 2004.

**Figure 5:**
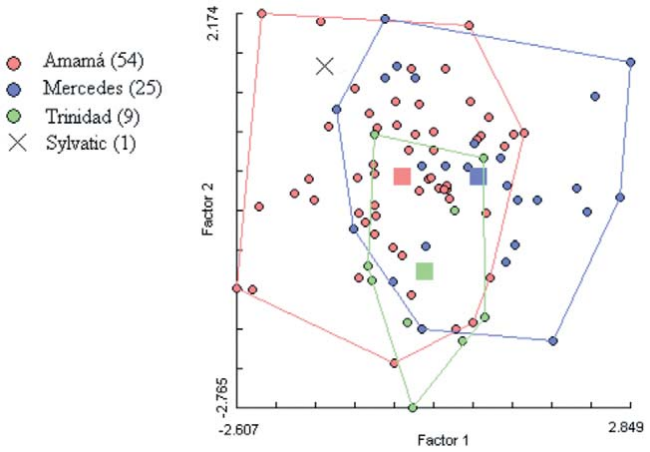
Factorial map of peridomestic and sylvatic *T. infestans* males using wing geometric morphometry.

Wing geometric morphometry was used to compare the only sylvatic *T. infestans* male collected (trap TN-139) with *T. infestans* males captured in local PD sites in 2002 and 2004. The factorial map showed that the sylvatic bug clearly overlapped with 2002 PD bugs from Amamá –the closest village to its capture site (Figure 5) and it was also assigned to 2004 PD bugs from Amamá (not shown).

### Spatial analyses

All sylvatic foci of *T. infestans* were located 110–2,300 m from the nearest D/PD sites ever found to be infested by this species after full-coverage insecticide spraying (i.e., detected during the preceding 18 months) (Figure 2). Trap location TN-182 included two *T. infestans*-positive sites (TN-180 and TN-182) that were analyzed together because their separation (13 m) was smaller than the distance resolution of the *G_i_(d)* test (50 m). The distance between traps positive for *T. infestans* to the nearest house varied from 125 to 1,900 m. Spatial analysis showed a statistically significant association (*G_i_(d)*>2.32, *P*<0.01) between two sylvatic foci of *T. infestans* found within three km of a D/PD site and the average timed-manual catch of bugs before insecticide spraying (Figure 2). Significant clustering occurred up to 1.2 km in Amamá (trap TN-139, with 17 insects) and up to 150 m in Mercedes (trap TN-101, with one third-instar nymph) (Figure 2). The remaining three sylvatic foci of *T. infestans* (TN-182, TN-180 and TN-92) were located at 430–1,846 m from the nearest infested house, but did not appear to be significantly associated with any of them (*G_i_(d)*>1.96; *P*>0.05).

## Discussion

We report here the first finding of multiple sylvatic foci of *T. infestans*: i) with normal chromatic characters (not “dark morphs”) in the Gran Chaco region outside Bolivia; ii) with morphological identification confirmed by DNA sequence information –ruling out taxonomic misdiagnosis of nymphs, and iii) with a genetic makeup indistinguishable from their local D/PD conspecifics in nearly all cases. The discovery of sylvatic foci of *T. infestans* was made possible by the extensive deployment of mouse-baited sticky traps in a wide diversity of habitats potentially suitable for the species as suggested by surveys in the Bolivian Chaco [43]. Although mouse-baited sticky traps may not achieve perfect detection of sylvatic foci [26], [71], the alternatives of using timed manual collections with a dislodging spray or habitat destruction are even less satisfactory or feasible [24]. Easy-to-use, more sensitive sampling methods for triatomine bugs in sylvatic habitats are crucially needed. Therefore, the actual prevalence of sylvatic foci of *T. infestans* as determined with mouse-baited traps was most likely underestimated.

Mitochondrial and microsatellite DNA markers coupled with wing geometric morphometry consistently indicated the occurrence of unrestricted gene flow between local D/PD and sylvatic *T. infestans* populations. In spite of the occurrence of private mitochondrial haplotypes and microsatellite alleles in sylvatic bugs, analyses suggest a strong genetic relationship with D/PD bugs. In the phylogenetic network, mtDNA sylvatic haplotypes were more frequently connected to peridomestic haplotypes rather than to other sylvatic variants ―which indicates that they did not form a population that evolved in isolation for a long period of time. A limitation here is that mtDNA only allows the estimation of historical female-based gene flow. However, microsatellite-based analyses ―a more suitable tool for detecting current gene flow― failed to reject that neighboring villages were the putative sources of sylvatic bugs in most cases. In addition, the small level of differentiation between sylvatic and D/PD specimens fell within the observed levels of within-population diversity [31], [60]. Therefore, there is no sufficient evidence to support restriction of gene flow between sylvatic and D/PD populations of *T. infestans* from the surrounding villages except in one case (trap TN-182).

Sibship analyses coupled with mitochondrial haplotype information at trap TN-139 over two trapping sessions separated by one month suggest that descendents from five different females were found at this rather remote site. Microsatellite data corroborated the heterogeneous genetic composition of TN-139 bugs as the insects were assigned to different reference populations. In the context of rare, light D/PD infestations after the insecticide spraying campaign, the finding of multiple haplotypes at a defined site was surprising. This sylvatic colony of *T. infestans* (the largest) was located 1.1 km away from the nearest infested house in an isolated habitat with no signs of current or past human use over the previous two decades (Figure S2). Moreover, another probable sylvatic foci of *T. infestans* with early-stage nymphs was detected in the vicinity of the largest sylvatic colony. Passive transport of *T. infestans* in the belongings of rural workers at a transitory camp may have provided an additional means of disseminating bugs within and between communities or the surrounding landscape. This alternative is worth considering because long-distance passive bug transport beyond its distribution range is well known and still occurs [72]. Thus, genetic and morphological evidence combined with the past history of denser D/PD infestations [8] suggests that the sylvatic specimens of *T. infestans* may have been feral derivatives (“spill-over”) of D/PD populations. Lack of sampling in sylvatic habitats before full-coverage insecticide spraying unfortunately does not allow establishing whether the sylvatic foci of *T. infestans* existed before or were formed as a consequence of flight dispersal of D/PD adult bugs or human-assisted passive transport of bugs.

These findings question the widely held notion of an unlikely continuous exchange of *T. infestans* bugs between wild and domestic habitats in the Chaco. Earlier studies using allozymes or morphometrics [20], [21] and mitochondrial DNA [32], [54] did not detect differences between sylvatic and domestic populations of *T. infestans* in the Andean Bolivian valleys, neither could mitochondrial markers in Chile [73]. In the allozyme-based study, the findings were interpreted as suggesting that sylvatic foci could be recent derivatives from nearby D/PD bug populations or vice versa –a pattern that was also consistent with unrestricted gene flow between domestic and sylvatic *T. cruzi* [20]. Intense gene flow between both types of bug populations (abundant at that time) could have generated the same patterns. Using head morphometry in the same study area in the Andean Bolivian valleys, reinfestant specimens of *T. infestans* found six months after house spraying with pyrethroids were considered survivors of the original domestic bug population unrelated to local sylvatic specimens [21]. Microsatellite data comparing the genetic makeup of sylvatic and D/PD populations of *T. infestans* showed restricted gene flow between sylvatic and peridomestic populations separated by only 300–650 m at 2,700 m altitude in the Andean Bolivian valleys [74], whereas they were highly structured and with evidence of low, asymmetric gene flow in a remote, well-preserved dry forest in the Argentinean Chaco [33]. Our data collected in highly-disturbed dry forest with more scattered houses show a different pattern and suggest that the occurrence of sylvatic foci of *T. infestans* may explain at least some of the new D/PD foci detected after full-coverage residual spraying of insecticides.

All sylvatic foci of *T. infestans* were 110–2,300 m from the nearest house or infested D/PD site detected after full-coverage insecticide spraying. These distances are within the estimated flight range of this species (1.5 km) derived from direct and indirect observations [12], [49], [50], [75], [76]. Because *T. infestans* may sustain tethered flights for >20 min at speeds of 2 m/s [77], the upper bound of its flight range may reach 3 km and remains uncertain. Therefore, the significant spatial associations detected combined with the range of distances between sylvatic and D/PD foci of *T. infestans* suggest that these habitats were probably connected through flight dispersal of adult bugs.

The identified habitats of sylvatic *T. infestans* in our study area were nearly all associated with trees at ground level, in fallen trees or tree stumps. No rocky outcrops were available. Potential bug refuges at higher altitude in the canopy ―difficult to spot and sample― were much less represented in our surveys. Compared with other sylvatic foci investigated with mouse-baited traps, the local apparent density of *T. infestans* (4 bugs per 100 trap-nights) was slightly higher than that recorded in remote dry forest in the Argentine Chaco (1.2 bugs per 100 trap-nights) [24], and substantially lower than in the Bolivian Chaco (17 bugs per 100 trap-nights) [43] or the Andean valleys (8–123 bugs per 100 trap-nights) [56]. The finding of small, malnourished sylvatic colonies with immature stages of *T. infestans* indicates that despite extensive deforestation and land-use change, the degraded forest still maintained suitable conditions and resources for bug development but at reduced levels: the apparent abundance and availability of blood-meal sources (not identified yet) in local sylvatic habitats was poorer and more unstable than in D/PD habitats. Some of the sylvatic bug foci in our study could be considered “semi-sylvatic”, in the sense that these habitats were intermediate between peridomestic ecotopes (such as pig or goat corrals made with piled thorny shrubs) and sylvatic habitats in terms of resident host species and abundance [46]. Semi-sylvatic habitats also tend to be less used and modified by regular human activities than peridomestic ecotopes. These findings suggest the possibility of sylvatic foci of *T. infestans* in almost any rural area within its geographic range. The domestication process *T. infestans* underwent in the past does not prevent the species from surviving at low density in a wide diversity of sylvatic or semi-sylvatic habitats, even after community-wide insecticide spraying.

Unlike previous reports in Argentina where the presence of sylvatic *T. infestans* may be a result of spill over from heavy D/PD infestations [reviewed in 24], the sylvatic *T. infestans* of this study occurred in sampling areas around villages under vector surveillance and selective control activities that only allowed the establishment of very few low density D/DP foci for limited time periods between surveys. A relevant question is whether the small-sized sylvatic bug populations we found are viable in the absence of immigration from D/PD sources (i.e., ‘rescue effects’) or they simply are temporary sinks. Our second-year follow-up data raise doubts about their viability over a longer time horizon in the absence of immigration, although removal of bugs may have contributed to apparent local extinctions. However, it is noteworthy that “dark morph” populations of *T. infestans* in the Bolivian and Argentine Chaco were viable despite having very low density and remote locations excluding them from D/PD ‘rescue effects’ [24], [25], [33].

*T. guasayana* was far more abundant than *T. infestans* in sylvatic habitats, and light-trap collections demonstrated the large number of flight-dispersing adult *T. guasayana*, as was found in the Bolivian and Paraguayan Chaco [78], [79]. Previous studies showed that *T. guasayana* also colonized peridomestic structures and semi-sylvatic ecotopes where it was associated positively with the local abundance of goats and the density of cacti and bromeliads [46]. Householders frequently collected adult bugs of this species when invading human habitations at sunset but this species was not able to colonize domestic premises before or after apparent suppression of *T. infestans* [8], [10], [46]. In the present study, the concurrent finding of *T. guasayana* in a fallen tree with fresh signs of *Didelphis* opossums suggests a close association with the main local sylvatic reservoir of *T. cruzi* typically infected with discrete typing unit I [42], [80]. The widespread occurrence and large abundance of *T. guasayana* combined with its ocassional infection, opportunistic blood-feeding behavior and dispersal ability implicate it as a secondary vector of *T. cruzi* in the peridomestic environment [45] and sylvatic habitats in the Argentine Chaco.

### Implications for vector control and elimination

A long-standing, key scientific question with vast implications for vector control is what is the source of the triatomine bugs appearing after community-wide insecticide spraying [18], [37], [81]. Are they (i) survivors or the offspring of previously existing bugs; (ii) immigrants from untreated D/PD or sylvatic foci; or (iii) migrants brought by passive transport from other villages or elsewhere? This issue is applicable to all major triatomine vector control programs throughout Latin America and the responses may differ between settings and even within the same species, as with *T. dimidiata* in Central America and Mexico or *T. brasiliensis* and *P. megistus* in Brazil –all of which display substantial within-species differences in habitat distribution, invasive capacity and other relevant traits. As with other species of triatomine bugs, *T. infestans* adults and nymphs are attracted to lights [48], [82]. Sylvatic populations of *T. infestans* are much more widespread than assumed in the past [23]–[29] and have recently been discovered in the Paraguayan Chaco [26]. Because sylvatic habitats are not targeted for vector control operations, they may provide hidden refuges for *T. infestans* from which they may reinvade houses in search of more suitable conditions and resources. Our results suggest that in areas with recurrent reinfestation, vector control programs should consider the potential occurrence of external sources (semi-sylvatic or sylvatic) around the target community. The role that sylvatic populations of *T. infestans* (either with melanic or normal phenotype) play in the process of recolonization of insecticide-treated villages and their invasive capacity needs to be more widely investigated to evaluate the risk they pose to effective vector control and eventual elimination in the Gran Chaco and elsewhere.

## Supporting Information

Figure S1 **Ecotopes where sylvatic foci of** ***T. infestans*** **were searched for and eventually detected.** A) holes of standing trees, B) dry cacti (*Opuntia quimilo* and *Opuntia ficus-indica*), C) terrestrial bromeliads (*Bromelia serra* and *Bromelia hieronymi*), D) piles of shrubs, E) tree trunks or stumps, F) holes of fallen trees.
(PDF)

Figure S2 **Two of the sylvatic foci where** ***T. infestans*** **was detected.** A) TN-139. B) TN-182.
(TIF)

Table S1 **Weight-to-length ratios of** ***T. infestans*** **and** ***T. guasayana*** **.** Mean for adult bugs, medians for nymphs, minimum and maximum values are reported according to collection site. Data for peridomestic bugs collected in Amamá, Trinidad and Mercedes (October 2000–August 2001) were taken from Ceballos et al. 2005 and L. A. Ceballos, unpublished data).
(DOC)

Table S2 **Mitochondrial haplotypes and microsatellite genotypes of sylvatic** ***T. infestans*** **.** NA: no PCR amplification. mtCOI Genbank accession numbers: EF451012-4, FJ811845, GQ478993, GQ478995, GQ478993. mtcytB Genbank accession numbers: AY062165, JN006793-9.
(DOC)

Table S3 **Sibship maximum likelihood analyses in traps TN-92 and TN-139.** Only values with probabilities greater than 0.6 are shown.
(DOC)

Table S4 **Reconstructed full- and half-sib families in TN-92 and TN-139.** A question mark means an unknown haplotype.
(DOC)

Alternative Language Abstract S1 **Translation of the abstract into Spanish by author Ricardo E. Gürtler.**
(DOC)
