# Supplementary figures and images for: Hidden Sylvatic Foci of the Main Vector of Chagas Disease Triatoma infestans: Threats to the Vector Elimination Campaign?
Source: PLoS Negl Trop Dis. 2011 Oct 25;5(10):e1365. doi: 10.1371/journal.pntd.0001365 (PMC3201917; doi:10.1371/journal.pntd.0001365)

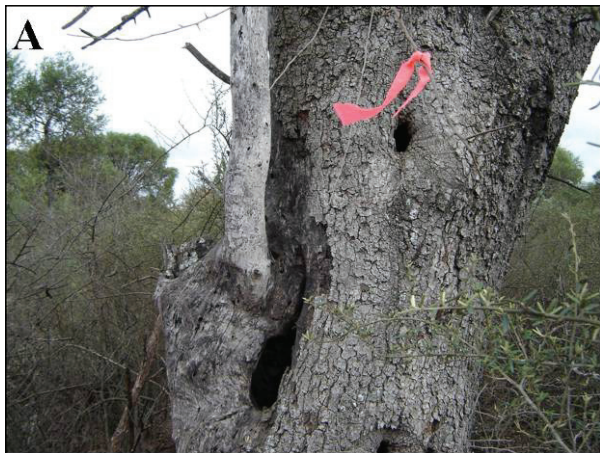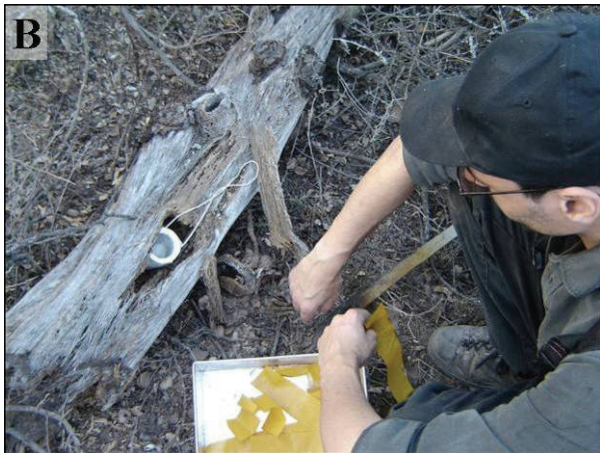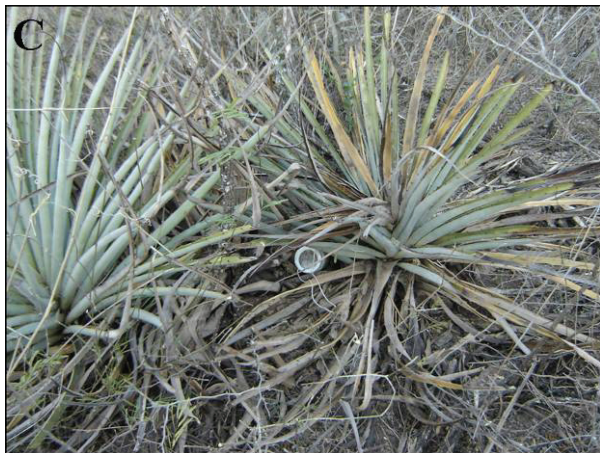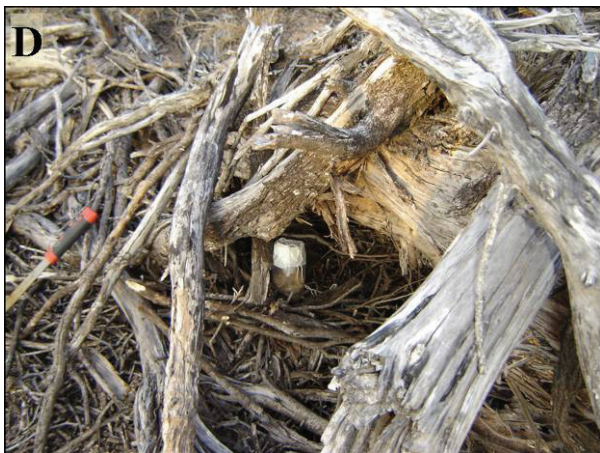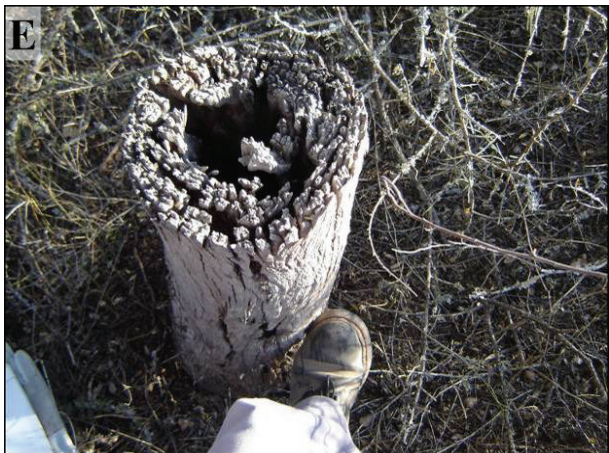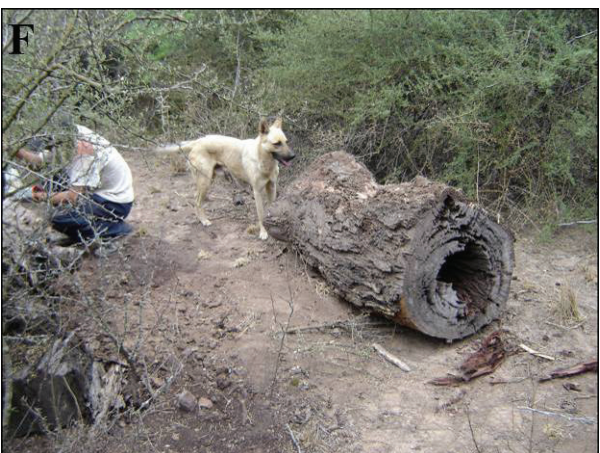

Supplement: Figure S1 — Ecotopes where sylvatic foci of T. infestans were searched for and eventually detected. A) holes of standing trees, B) dry cacti (Opuntia quimilo and Opuntia ficus-indica), C) terrestrial bromeliads (Bromelia serra and Bromelia hieronymi), D) piles of shrubs, E) tree trunks or stumps, F) holes of fallen trees. (PDF) [file pntd.0001365.s001.pdf]

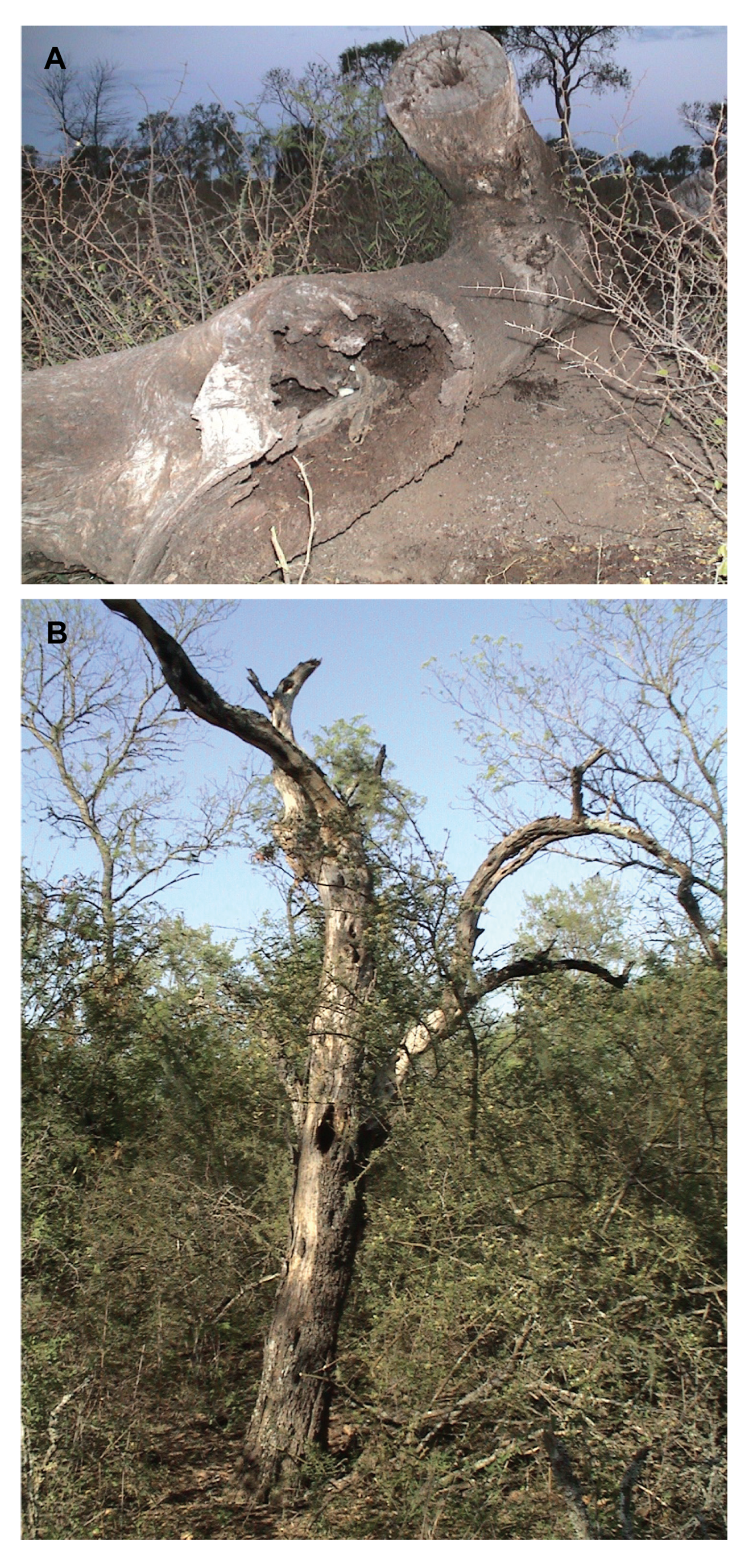

Supplement: Figure S2 — Two of the sylvatic foci where T. infestans was detected. A) TN-139. B) TN-182. (TIF) [file pntd.0001365.s002.tif]
