# Supplementary material for: Hidden Sylvatic Foci of the Main Vector of Chagas Disease Triatoma infestans: Threats to the Vector Elimination Campaign?
Source: PLoS Negl Trop Dis. 2011 Oct 25;5(10):e1365. doi: 10.1371/journal.pntd.0001365 (PMC3201917; doi:10.1371/journal.pntd.0001365)
Supplement: Table S1 — Weight-to-length ratios of T. infestans and T. guasayana . Mean for adult bugs, medians for nymphs, minimum and maximum values are reported according to collection site. Data for peridomestic bugs collected in Amamá, Trinidad and Mercedes (October 2000–August 2001) were taken from Ceballos et al. 2005 and L. A. Ceballos, unpublished data). (DOC) [file pntd.0001365.s003.doc]

| Collection  site |  | *T. infestans* | | | | *T. guasayana* | | | |
| --- | --- | --- | --- | --- | --- | --- | --- | --- | --- |
|  | Stage | No.  examined | W/L | Min. | Max. | No.  examined | W/L | Min. | Max. |
| Sylvatic |  |  |  |  |  |  |  |  |  |
|  | Male | 1 | 4.99 | - | - | 31 | 2.86 | 1.88 | 4.21 |
|  | Female | 0 | - | - | - | 72 | 3.38 | 2.19 | 9.04 |
|  | Nymph V | 1 | 5.01 | - | - | 17 | 4.58 | 2.24 | 8.52 |
|  | Nymph IV | 5 | 2.37 | 1.92 | 3.06 | 2 | 2.31 | 1.40 | 3.22 |
| Peridomestic | |  |  |  |  |  |  |  |  |
|  | Male | 365 | 9.60 | 2.91 | 16.57 | 14 | 5.10 | 3.10 | 9.47 |
|  | Female | 184 | 11.49 | 4.80 | 26.50 | 10 | 8.22 | 5.08 | 12.64 |
|  | Nymph V | 162 | 8.70 | 1.90 | 20.23 | 64 | 8.74 | 2.55 | 20.65 |
|  | Nymph IV | 83 | 3.31 | 1.19 | 10.22 | 13 | 3.13 | 0.53 | 6.76 |
