# Supplementary material for: Hidden Sylvatic Foci of the Main Vector of Chagas Disease Triatoma infestans: Threats to the Vector Elimination Campaign?
Source: PLoS Negl Trop Dis. 2011 Oct 25;5(10):e1365. doi: 10.1371/journal.pntd.0001365 (PMC3201917; doi:10.1371/journal.pntd.0001365)
Supplement: Table S2 — Mitochondrial haplotypes and microsatellite genotypes of sylvatic T. infestans . NA: no PCR amplification. mtCOI Genbank accession numbers: EF451012-4, FJ811845, GQ478993, GQ478995, GQ478993. mtcytB Genbank accession numbers: AY062165, JN006793-9. (DOC) [file pntd.0001365.s004.doc]

| Insect | Trap TN | Tims3 | Tims5 | Tims19 | Tims22 | Tims23 | Tims27 | Tims42 | Tims56 | Tims64 | Tims65 | COI | cytB |
| --- | --- | --- | --- | --- | --- | --- | --- | --- | --- | --- | --- | --- | --- |
| SIL-1 | 92A | 200/202 | 363/388 | 345/371 | 174/194 | 160/162 | 289/301 | 220/220 | 157/161 | 145/145 | 231/239 | d | XXXVI |
| SIL-2 | 92 | 200/200 | 377/392 | 343/343 | 174/176 | 160/162 | 289/301 | 220/220 | 157/161 | 147/147 | 231/239 | d | XXXVI |
| SIL-5 | 92 | 200/202 | 363/388 | 344/371 | 174/176 | 160/162 | 289/305 | 220/220 | 155/161 | 145/145 | 231/239 | d | XXXVI |
| SIL-3 | 101 | 183/200 | 388/396 | 334/334 | 174/192 | 162/170 | 291/299 | 210/218 | 159/161 | 147/147 | 239/239 | e | VIII |
| SIL-6 | 139 | 183/200 | 379/380 | 350/356 | 174/176 | 162/170 | 295/301 | 222/229 | 161/163 | 147/149 | 231/241 | c | II |
| SIL-14 | 139 | 200/200 | 380/400 | 350/375 | 168/198 | 162/162 | 289/301 | 201/215 | 157/157 | 147/147 | 241/243 | ao | XXXVII |
| SIL-15 | 139 | 200/200 | 373/390 | 350/375 | 168/198 | 160/162 | 289/301 | 205/239 | 159/159 | 147/147 | 241/243 | ao | XXXVII |
| SIL-30 | 139 | 200/200 | 380/390 | 350/356 | 188/190 | 162/162 | 289/305 | 206/218 | 159/161 | 147/147 | 207/207 | NA | NA |
| SIL-31 | 139 | 200/200 | 375/390 | 346/358 | 174/176 | 160/162 | 289/301 | 201/218 | 159/163 | 147/147 | 235/247 | NA | V |
| SIL-32 | 139 | 200/200 | 375/390 | 346/358 | 174/190 | 162/162 | 289/301 | 201/224 | 159/159 | 147/147 | 223/223 | c | V |
| SIL-33 | 139 | 183/200 | 382/390 | 350/350 | 174/180 | 162/162 | 289/289 | 210/218 | 159/159 | 147/147 | 249/249 | am | XIV |
| SIL-34 | 139 | 200/204 | 382/390 | 350/350 | 180/190 | 162/162 | 289/289 | 201/206 | 159/159 | 149/149 | 233/233 | am | XIV |
| SIL-35 | 139 | 200/200 | 379/382 | 346/358 | 174/190 | 160/162 | 289/301 | 201/224 | 159/159 | 147/147 | 223/223 | c | V |
| SIL-36 | 139 | 200/200 | 380/390 | 350/358 | 168/190 | 162/162 | 289/291 | 201/206 | 159/159 | 147/147 | 243/247 | aq | I |
| SIL-37 | 139 | 200/200 | 390/390 | 356/358 | 168/176 | 162/162 | 289/291 | 206/218 | 159/159 | 147/147 | 243/243 | aq | NA |
| SIL-38 | 139 | 183/200 | 382/390 | 350/356 | 174/180 | 162/162 | 289/289 | 201/206 | 157/157 | 147/147 | 248/248 | am | XIV |
| SIL-39 | 139 | 200/200 | 380/382 | 358/358 | 188/190 | 162/162 | 289/291 | 214/218 | 157/157 | 147/147 | 207/207 | aq | I |
| SIL-40 | 139 | 200/200 | 380/382 | 350/356 | 168/190 | 160/162 | 289/305 | 214/218 | 157/157 | 147/147 | 243/243 | aq | I |
| SIL-41 | 139 | NA | NA | NA | NA | NA | NA | NA | NA | NA | NA | am | NA |
| SIL-42 | 139 | 200/200 | 380/382 | 356/358 | 168/174 | 162/162 | 289/305 | 201/214 | 157/157 | 147/147 | 243/247 | aq | NA |
| SIL-43 | 139 | 200/200 | 380/382 | 356/358 | 188/190 | 162/162 | 289/291 | 201/206 | 159/159 | 147/147 | 207/207 | NA | NA |
| SIL-12 | 182 | 183/204 | 373/384 | 343/343 | 180/194 | 160/162 | 297/311 | 218/231 | 159/165 | 150/150 | 233/252 | al | VII |
