# Supplementary material for: Hidden Sylvatic Foci of the Main Vector of Chagas Disease Triatoma infestans: Threats to the Vector Elimination Campaign?
Source: PLoS Negl Trop Dis. 2011 Oct 25;5(10):e1365. doi: 10.1371/journal.pntd.0001365 (PMC3201917; doi:10.1371/journal.pntd.0001365)
Supplement: Table S3 — Sibship maximum likelihood analyses in traps TN-92 and TN-139. Only values with probabilities greater than 0.6 are shown. (DOC) [file pntd.0001365.s005.doc]

| Trap | Relationship | Offspring 1 | Offspring 2 | Probability |
| --- | --- | --- | --- | --- |
| TN-92 | Full-sibs | SIL-1 | SIL-5 | 0.995 |
|  | Half-sibs | SIL-1 | SIL-2 | 0.798 |
|  |  | SIL-2 | SIL-5 | 0.801 |
| TN-139 | Full-sibs | SIL-32 | SIL-35 | 1.000 |
|  |  | SIL-14 | SIL-15 | 0.989 |
|  |  | SIL-36 | SIL-43 | 0.958 |
|  |  | SIL-36 | SIL-42 | 0.951 |
|  |  | SIL-42 | SIL-43 | 0.921 |
|  |  | SIL-31 | SIL-32 | 0.850 |
|  |  | SIL-31 | SIL-35 | 0.850 |
|  |  | SIL-39 | SIL-40 | 0.839 |
|  |  | SIL-30 | SIL-40 | 0.823 |
|  |  | SIL-30 | SIL-39 | 0.804 |
|  |  | SIL-33 | SIL-38 | 0.754 |
|  |  | SIL-34 | SIL-38 | 0.667 |
|  | Half-sibs | SIL-37 | SIL-42 | 0.969 |
|  |  | SIL-36 | SIL-37 | 0.950 |
|  |  | SIL-37 | SIL-43 | 0.934 |
|  |  | SIL-37 | SIL-39 | 0.899 |
|  |  | SIL-37 | SIL-40 | 0.874 |
|  |  | SIL-30 | SIL-37 | 0.846 |
|  |  | SIL-2 | SIL-5 | 0.801 |
|  |  | SIL-1 | SIL-2 | 0.798 |
|  |  | SIL-36 | SIL-40 | 0.647 |
|  |  | SIL-40 | SIL-43 | 0.641 |
|  |  | SIL-30 | SIL-42 | 0.639 |
|  |  | SIL-40 | SIL-42 | 0.603 |
|  |  | SIL-30 | SIL-36 | 0.600 |
