# Supplementary material for: Hidden Sylvatic Foci of the Main Vector of Chagas Disease Triatoma infestans: Threats to the Vector Elimination Campaign?
Source: PLoS Negl Trop Dis. 2011 Oct 25;5(10):e1365. doi: 10.1371/journal.pntd.0001365 (PMC3201917; doi:10.1371/journal.pntd.0001365)
Supplement: Table S4 — Reconstructed full- and half-sib families in TN-92 and TN-139. A question mark means an unknown haplotype. (DOC) [file pntd.0001365.s006.doc]

| Trap | Half-sib family | Full-sib family | Members | | | *mtCOI-cytb* haplotype |
| --- | --- | --- | --- | --- | --- | --- |
| TN-92 | 1 | 1-1 | SIL-1 | SIL-5 |  | d-XXXVI |
|  |  | 1--2 | SIL-2 |  |  | d-XXXVI |
| TN-139 | 1 | 1-1 | SIL-14 | SIL-15 |  | ao-XXXVII |
|  | 2 | 2-1 | SIL-31 | SIL-32 | SIL-35 | c-V, ?-V |
|  | 3 | 3-1 | SIL-36 | SIL-42 | SIL-43 | aq-I, aq-?, ?-? |
|  | 3 | 3-2 | SIL-30 | SIL-39 | SIL-40 | aq-I, ?-? |
|  | 3 | 3-3 | SIL-37 |  |  | aq-? |
|  | 4 | 4-1 | SIL-33 | SIL-34 | SIL-38 | am-XIV |
|  | 5 | 5-1 | SIL-6 |  |  | c-II |
