# Supplementary material for: Hidden Sylvatic Foci of the Main Vector of Chagas Disease Triatoma infestans: Threats to the Vector Elimination Campaign?
Source: PLoS Negl Trop Dis. 2011 Oct 25;5(10):e1365. doi: 10.1371/journal.pntd.0001365 (PMC3201917; doi:10.1371/journal.pntd.0001365)
Supplement: Alternative Language Abstract S1 — Translation of the abstract into Spanish by author Ricardo E. Gürtler. (DOC) [file pntd.0001365.s007.doc]

**Alternative Language Translation S1.** Translation of the abstract into Spanish by author Ricardo E. Gürtler.

Resumen

*Triatoma infestans*, una especie altamente domesticada e históricamente el principal vector de la enfermedad de Chagas, es el objetivo de un programa de eliminación basado en la aplicación de insecticidas en los países del cono sur de Sudamérica desde 1991. Solo se han alcanzado éxitos limitados en la región del Gran Chaco debido a reinfestaciones repetidas. Llevamos a cabo un rociado con insecticidas piretroides de todas las viviendas de un área bien definida en el noroeste de Argentina, seguido por un monitoreo intenso de la reinfestación de esas viviendas y de búsquedas de triatominos ("vinchucas") en hábitats silvestres durante los siguientes dos años, para establecer las posibles fuentes de las nuevas colonias en las viviendas. Hallamos seis focos silvestres de *T. infestans* con baja densidad en árboles situados dentro del rango de vuelo de la especie respecto a la vivienda infestada más cercana que habíamos detectado antes de las intervenciones de control. Usando múltiples métodos (imágenes satelitales de alta resolución, sistemas de información geográfica, estadística espacial, marcadores genéticos y morfometría geométrica de alas), corroboramos la identidad específica de las vinchucas silvestres como *T. infestans* y hallamos que eran indistinguibles de, o estaban muy estrechamente relacionadas con las poblaciones locales de vinchucas halladas en hábitats domésticos o peridomésticos. Dos focos silvestres estaban asociados significativamente con las poblaciones de vinchucas más cercanas detectadas antes de las intervenciones de control. Las hábitats silvestres albergan focos ocultos de *T. infestans* que pueden representar una amenaza a los esfuerzos de eliminación del vector.
